# Specificity and Plasticity of Thalamocortical Connections in *Sema6A* Mutant Mice

**DOI:** 10.1371/journal.pbio.1000098

**Published:** 2009-04-28

**Authors:** Graham E Little, Guillermina López-Bendito, Annette E Rünker, Noelia García, Maria C Piñon, Alain Chédotal, Zoltán Molnár, Kevin J Mitchell

**Affiliations:** 1 Smurfit Institute of Genetics, Trinity College Dublin, Dublin, Ireland; 2 Department of Physiology, Anatomy and Genetics, University of Oxford, Oxford, Oxfordshire, United Kingdom; 3 Instituto de Neurociencias de Alicante, CSIC & Universidad Miguel Hernández, San't Joan d'Alacant, Spain; 4 INSERM, UMR S968, Institut de la Vision, Department of Development, Paris, France; 5 UPMC Univ Paris 06, UMR S968, Institut de la Vision, Paris, France; Cambridge University, United Kingdom

## Abstract

The establishment of connectivity between specific thalamic nuclei and cortical areas involves a dynamic interplay between the guidance of thalamocortical axons and the elaboration of cortical areas in response to appropriate innervation. We show here that *Sema6A* mutants provide a unique model to test current ideas on the interactions between subcortical and cortical guidance mechanisms and cortical regionalization. In these mutants, axons from the dorsal lateral geniculate nucleus (dLGN) are misrouted in the ventral telencephalon. This leads to invasion of presumptive visual cortex by somatosensory thalamic axons at embryonic stages. Remarkably, the misrouted dLGN axons are able to find their way to the visual cortex via alternate routes at postnatal stages and reestablish a normal pattern of thalamocortical connectivity. These findings emphasize the importance and specificity of cortical cues in establishing thalamocortical connectivity and the spectacular capacity of the early postnatal cortex for remapping initial sensory representations.

## Author Summary

During brain development, the emergence of distinct areas in the cerebral cortex involves an interplay between patterning of the cortical sheet in the early embryo and later influences of incoming connections made from other brain areas, namely the thalamus. Connectivity between the thalamus and the cortex is initially smooth and graded, and a prominent model for how thalamocortical connectivity is established proposes thalamic axons are topographically sorted as they course through subcortical regions and then passively delivered to appropriate areas of the cortical sheet. We have used mutant mice lacking the guidance molecule *Semaphorin-6A* to test this model. In these mutants, *Semaphorin-6A* axons from the visual part of the thalamus are subcortically misrouted and fail to innervate the presumptive visual cortex, which is instead invaded by somatosensory thalamic axons. Despite this major disruption in initial connectivity, many visual thalamic axons find their way specifically to visual cortex, arriving several days later than usual. These late-arriving axons often follow alternate routes, and upon arrival are able to out-compete earlier-arriving somatosensory axons to reestablish grossly normal thalamocortical connectivity. These results argue strongly against an essential role for early subcortical targeting in the establishment of thalamocortical connectivity patterns and suggest instead the existence of highly specific target-selection mechanisms that match thalamic axons with appropriate cortical areas.

## Abstract 3

Initial misrouting and subsequent recovery of thalamocortical axon projections in Semaphorin-6A mutant mice highlights the importance of specific target-selection mechanisms that match thalamic axons with appropriate cortical areas.

## Introduction

A dynamic interplay exists between the processes of cortical arealization and those controlling the guidance and targeting of thalamocortical projections [1–5]. Early in development, both the thalamic field and the cortical sheet appear homogeneous in cytoarchitecture, and connections between them form in a smoothly topographic fashion, with dorsolateral thalamus projecting to caudal cortex and ventromedial thalamus to rostral cortex [6–8]. The cytoarchitectonic resolution of these fields into discrete cortical areas and thalamic nuclei occurs later [8–13] with the elaboration of many aspects of the cortical areas dependent on appropriate thalamic innervation [1–5,14,15].

Several lines of evidence have led to the theory that subcortical sorting of thalamic axons within the ventral telencephalon largely determines their final targeting within the cortex [16–20]. For example, in mutants in the transcription factor *Ebf1* or in the *Dlx1/Dlx2* double mutants, a subset of thalamic axons is misrouted ventrally, resulting in a caudal shift of the remaining axons within the ventral telencephalon [16]. This shift is projected onto the cortex so that at birth, caudal cortical areas are contacted by axons that would normally project to more rostral areas. The ultimate effect of this derangement on thalamocortical connectivity could not be assessed in these mutants, however, as they die perinatally.

On the other hand, many experiments have revealed the existence of cortex-specific cues that control thalamocortical targeting [21–27]. For example, changes in patterning of the cortical sheet in *Emx2* [21,24], *Fgf8* [23], or *COUP-TF1* [27] mutants lead to parallel alterations in the patterns of thalamocortical connectivity. In each of these cases, manipulations solely in the cortex dramatically affect thalamocortical targeting. Indeed, ectopic expression of Fgf8 in either the subplate or cortical plate further revealed that thalamocortical axons (TCAs) are responsive to guidance cues present in both the subplate and cortical plate [26]. The interplay between subcortical and cortical mechanisms in determining eventual thalamocortical connectivity thus remains to be resolved.

To get a better understanding of the interactions between areal patterning and thalamic axon guidance, we have used the *Sema6A* gene trap mouse. As a consequence of *Sema6A* disruption, a large fraction of thalamic projections gets derailed within the ventral telencephalon [28]. As these mice survive to adulthood they provide a unique model with normal cortical patterning but altered thalamic input during embryonic life. Our study reveals a changing pattern of thalamocortical development in the *Sema6A* mutants, drawing attention to the spectacular capacity of the cortex for altering and organizing its initial sensory representation. In particular, our findings suggest that thalamic axons from the dorsal lateral geniculate nucleus (dLGN) can target their correct area even if they arrive there days later than normal, through alternate subcortical routes. They also indicate that dLGN axons can out-compete invading axons from inappropriate thalamic nuclei, establishing a surprisingly normal adult cortical representation.

## Results

### *Sema6A* Is Strongly Expressed in the Developing Thalamus and Ventral Telencephalon

**Figure 1:**
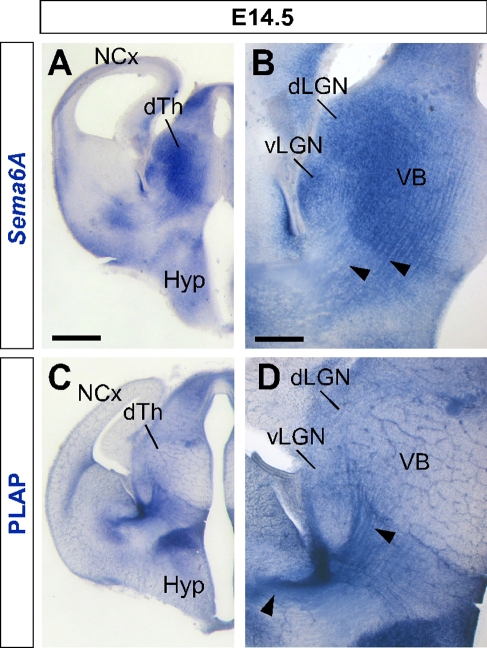
*Sema6A* Is Highly Expressed in the Developing Embryonic Thalamus (A and B) Coronal section of an E14.5 mouse brain showing in situ hybridization for *Sema6A* in the forebrain (A). *Sema6A* is expressed in neurons and axons (arrowheads) of the dorsal geniculate nucleus (dLGN) and ventrobasal complex (VB) of the developing dorsal thalamus (dTh; [B]). (C and D) PLAP staining on comparable coronal sections of *Sema6A* ^+/−^ mouse brains. PLAP-positive axons, presumably TCAs, can be seen projecting from the dTh through the internal capsule towards the neocortex (NCx; arrowheads). Hyp, hypothalamus; vLGN, ventrolateral geniculate nucleus. Scale bars in (A and C) indicate 500 μm; in (B and D) 200 μm.

*Sema6A* is broadly expressed in the thalamus at embryonic day (E)14.5, a time when thalamic neurons are extending axons towards their cortical targets [28], with expression highest in the dorsolateral aspect (*n* = 5; Figure 1A and 1B). *Sema6A* is also strongly expressed in the amygdala and the ventral telencephalon, and weakly expressed in the neocortex at this age, localized to the most superficial compartments (Figure 1A and 1B). At late embryonic stages, *Sema6A* is also expressed by deep cortical plate neurons, eventually layer 5 (unpublished data). Staining with the axonal marker placental alkaline phosphatase (PLAP), encoded on the gene trap cassette in this *Sema6A* allele, revealed thalamic axons extending from the thalamus through the internal capsule and towards the neocortex (Figure 1C and 1D).

### Lack of *Sema6A* Leads to Abnormalities in Thalamocortical Pathfinding

**Figure 2:**
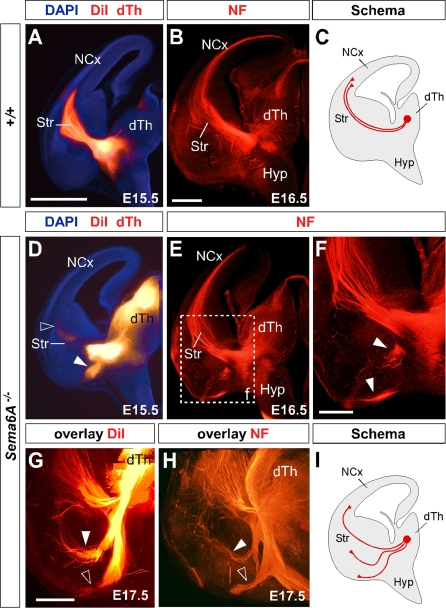
Early Thalamocortical Guidance Defects in *Sema6A^−/−^* Mouse Coronal sections showing half the brain or close-ups of ventral telencephalon. (A and B) TCAs follow a normal route along the ventral telencephalon and striatum (Str) in wild-type embryos as revealed by DiI tracing from the dorsal thalamus (dTh, [A]) as well as by neurofilament staining (B). (D–H) In *Sema6A^−/−^* embryos, a large proportion of TCAs are derailed at the ventral telencephalon (filled arrowheads in [D and H]), as shown by DiI tracing from the internal capsule (ic) at E15.5 (D) and neurofilament staining at E16.5 (E and F), and an overlaid series of images at E17.5 (G and H). Note that some TCAs follow their normal route through the striatum (Str) in the *Sema6A^−/−^* brains (open arrowhead in [D]). Also, note that misrouted TCAs in the *Sema6A^−/−^* brains appear to bifurcate in the ventral telencephalon (open and filled arrowheads in [H]). (C and I) Schematic diagrams of the trajectory of TCAs in wild-type (C) and *Sema6A^−/−^* brains (I). Scale bars in (A and D) indicate 1 mm; in (B and E) 500 μm, in (F) 300 μm; and in (G and H) 250 μm. Hyp, hypothalamus; NCx, neocortex; Str, striatum.

A previous study using PLAP staining showed that many thalamic axons were misrouted in the *Sema6A^−/−^* brains at embryonic stages [28]. To further examine the guidance of TCAs in the absence of functional Sema6A protein, we performed carbocyanine dye tracing studies. Broad injections of DiI in the thalamus (including the dorsolateral aspect) at E15.5 revealed a prominent derailment of thalamic axons at the surface of the ventral telencephalon and amygdala in *Sema6A^−/−^* embryos (*n* = 4/4; Figure 2D and 2G–2I), compared to the normal route of navigation through the internal capsule towards the neocortex observed in wild-type embryos (*n* = 4/4; Figure 2A and 2C). The derailment of a large proportion of TCA fibers at the ventral telencephalon in *Sema6A^−/−^* brains at E16.5 was confirmed by neurofilament (NF) immunohistochemistry (*n* = 3; Figure 2E and 2F) and by PLAP staining (Figure S1A and S1C). Overlaid consecutive serial sections of *Sema6A^−/−^* brains at E17.5 stained for NF reveal more clearly the extent of the TCA derailment (*n* = 3; Figure 2H).

### Only dLGN Axons Are Misrouted within the Ventral Telencephalon

**Figure 3:**
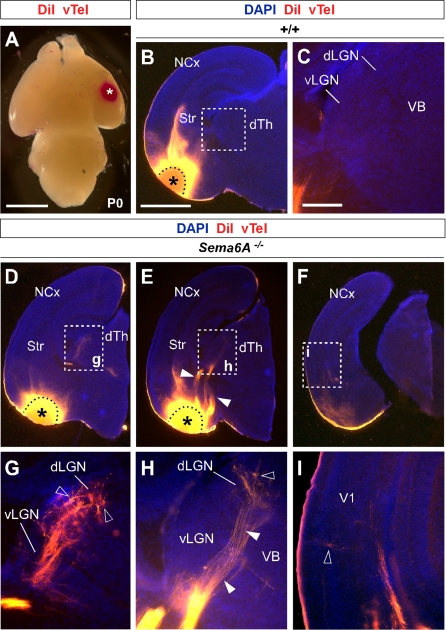
Visual Thalamocortical Neurons Misproject to the Ventral Telencephalon in the Absence of Sema6A Function (A) DiI crystals were placed at the surface of the ventral telencephalon in wild-type and *Sema6A^−/−^* brains at P0 (ventral view). All other panels are coronal sections. (B and C). No back-labeled cells were seen in the dorsal thalamus (dTh) after ventral telencephalic DiI placements in wild-type brains. (D–I) Rostrocaudal coronal sections of a *Sema6^ −/−^* brain showing abnormal axonal projections (filled arrowheads in [E and H]) from the ventral telencephalon to the dTh. Many back-labeled cells were observed located at the dorsal lateral geniculate nucleus (dLGN; open arrowheads in [G and H]). At caudal levels, some back-labeled cells were located at the primary visual cortex (V1; open arrowhead in [I]). Asterisks in (A, B, D, and E) indicate the DiI crystal placement sites. Scale bars in (A) indicate 2.5 mm; in (B and D–F) 1 mm; and in (C and G–I) 200 μm. Str, striatum; VB, ventrobasal nucleus; vLGN, ventral lateral geniculate nucleus.

To identify the precise origin of the misrouted thalamic axons within the thalamus, we placed small DiI crystals at the site of the derailed fibers near the ventral surface of the telencephalon (Figure 3A) in wild-type (*n* = 4) and *Sema6A^−/−^* (*n* = 4) postnatal day (P)0 brains. Whereas no back-labeled cells were observed in any thalamic nuclei in wild-type brains (Figure 3B and 3C), several axon bundles were retrogradely traced to the dorsolateral aspect of the thalamus in *Sema6A^−/−^* brains (Figure 3D and 3E). Retrogradely labeled cells were specifically found in the presumptive dLGN (Figure 3G and 3H). Some labeled bundles were also observed ascending laterally towards the cortex (Figure 3E). Moreover, in *Sema6A^−/−^* brains, at more-caudal telencephalic levels, DiI-labeled axons were observed running through the intermediate zone of the primary visual cortical area (Figure 3F and 3I; and unpublished data), suggesting that some dLGN axons that follow this abnormal route might still reach the visual cortex. Indeed, whereas a DiA crystal placed in the internal capsule zone of wild-type brains at E17.5 back-labeled cells throughout the dorsal thalamus (*n* = 2/2; Figure S2A–S2C), a similarly placed DiA crystal in *Sema6A^−/−^* brains at the same age strikingly did not label cells in the dLGN (*n* = 2/2; Figure S2D–S2F).

**Figure 4:**
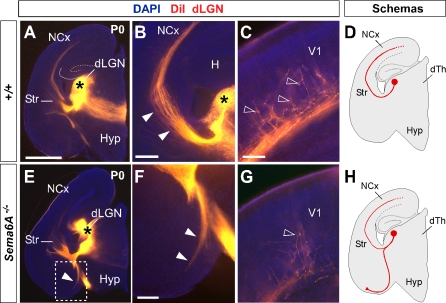
DiI Crystal Placements to the Dorsal Lateral Geniculate Nucleus (dLGN) Confirmed That Visual Thalamic Axons Are Severely Affected by the Absence of Sema6A Function Coronal sections showing half the brain or close-ups of specific areas. (A–H) Small crystals of DiI were implanted into the dLGN of wild-type (A–D) and *Sema6A^−/−^* (E–H) brains at P0. Asterisks in (A, B, and E) indicate the DiI crystal placement sites. (A–D) DiI injections in the dLGN of wild-type brains, labeled thalamic axons extending through the internal capsule and striatum (Str), and entering to the neocortex (NCx; filled arrowheads in [B]). Many back-labeled cells were seen at the primary visual cortex (V1, open arrowheads in [C]). (E–H) DiI placements in the dLGN of *Sema6A^−/−^* brains labeled bundles of axons misrouted at the ventral telencephalon (filled arrowheads in [E and F]). Fewer back-labeled cells were observed at V1 in *Sema6A^−/−^* brains (open arrowhead in [G]) compared to control. Scale bars in (A) indicate 3 mm; in (B) 1 mm; in (C and G) 100 μm; and (F) 200 μm. H, hippocampus; Hyp, hypothalamus.

To further study the trajectories of the dLGN projections, we placed small DiI crystals into the dLGN of wild-type and *Sema6A^−/−^* brains at P0. At this age, wild-type and heterozygous dLGN axons extended through the internal capsule and arrived to the visual cortex in a normal fasciculation pattern (*n* = 8/8; Figure 4A–4D). In *Sema6A^−/−^* brains, in contrast, dLGN axons projected ventrally to the most superficial regions of the ventral telencephalon. Despite this, a small number of dLGN axon terminals were observed in visual cortex (Figure 4G; *n* = 9/9). These results demonstrate that the vast majority of visual TCAs are severely affected by the lack of Sema6A function in vivo, but some still reach presumptive visual cortex by birth, possibly by alternate routes.

### The Early Topography of TCAs Is Altered in *Sema6A^−/−^* Mice

**Figure 5:**
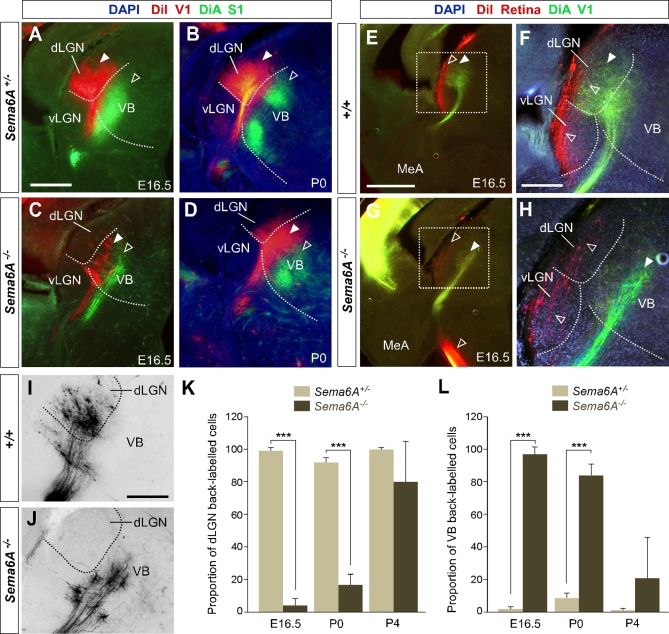
Early Topographical Defects in Thalamocortical Connectivity in *Sema6A^−/−^* Mice (A–J) Coronal sections showing back-labeling in dorsal thalamus. (A and B) In *Sema6A^+/−^* brains, DiI crystal placement in the occipital cortex back-labels cells in the dorsal lateral geniculate nucleus (dLGN; filled arrowheads), whereas a DiA crystal placement in parietal cortex back-labels cells in the ventrobasal complex (VB; open arrowheads). (C–D) In *Sema6A^−/−^* brains, identical dye crystal placements resulted in back-labeled red (DiI) and green (DiA) cells in the VB of the dorsal thalamus, indicating a medial shift of visual thalamocortical connectivity in these mice. Note that at E16.5 (C), the visual shift was more pronounced than at P0 stages (D). (E–H) In *Sema6A^+/−^* brains at E16.5, retinothalamic projections, labeled by a DiI crystal placed in the retina, entered the ventral and dorsal lateral geniculate nucleus (vLGN and dLGN, respectively; open arrowheads in [E and F]). A DiA crystal placement in the occipital cortex back-labeled dLGN cells (filled arrowheads in [E and F]). In contrast, in *Sema6A^−/−^* brains, whereas the retinal projection enters the vLGN and dLGN (open arrowheads [G and H]) normally, thalamic cells in the VB are back-labeled from a DiA crystal in the occipital cortex (filled arrowheads, [G and H]). (I–L) At early stages (E16.5), the vast majority of cells back-labeled from the occipital cortex are found in the dLGN in *Sema6A^+/−^* mice, with few if any, being found in the VB. In *Sema6A^−/−^* mice at the same age, the majority of cells back-labeled from the occipital cortex are found in the VB, with very few being found in the dLGN. These differences are highly statistically significant (triple asterisks [***] indicates *p* <0.0001). At P0, the proportion of cells back-labeled from the occipital cortex and found in the dLGN is significantly greater than that at E16.5 but still significantly less than in *Sema6A^+/−^* mice (*p* < 0.0001). By P4, there was no significant difference between wild-type and *Sema6A^−/−^* mice in the proportion of cells back-labeled from the occipital cortex and found in the dLGN. Error bars indicate standard error mean (s.e.m.). Scale bars in (A–D) indicate 200 μm; in (E and G) 200 μm; in (F and H) 200 μm; and in (I and J) 100 μm. MeA, medial amygdala.

To determine whether the initial derailment of dLGN axons could affect the general topographical arrangement between neocortex and thalamus, we performed multiple cortical dye placements at E16.5 and P0. We placed DiI and DiA crystals into the putative visual and somatosensory cortices, respectively, in *Sema6A^+/−^* and *Sema6A^−/−^* mutant brains (Figure 5). In *Sema6A^+/−^* animals (*n* = 6/6 E16.5; *n* = 5/5 P0), a DiI crystal placement in the occipital neocortex labeled thalamic cell bodies and cortical axons in the dLGN (Figure 5A and 5B). Placements of DiA in the parietal neocortex labeled cells and axon terminals in a more medial thalamic domain, where the ventrobasal (VB) complex is located (Figure 5A and 5B). Interestingly, similar placements performed in *Sema6A^−/−^* embryos showed a lateral to medial shift in the thalamocortical and reciprocal corticothalamic connectivity (*n* = 8/8 E16.5; *n* = 5/5 P0; Figure 5C and 5D). Specifically, placements into the occipital cortex labeled large numbers of cells in the lateral part of VB in *Sema6A^−/−^* brains, whereas few, if any cells at E16.5 were back-labeled in the dLGN (Figure 5I–5L; 96.56 ± 4.6% of thalamic cells back-labeled from occipital cortex in E16.5 *Sema6A^−/−^* brains were found in the VB, compared to just 1.25 ± 2.25% of back-labeled thalamic cells in *Sema6A^+/−^* brains, *p* < 0.0001). At P0, an increase in the number of cells back-labeled in dLGN was observed in the mutants, though this was still far fewer than in wild-type animals (Figure 5K and 5L; 16.24 ± 6.9% of thalamic cells back-labeled from occipital cortex in P0 *Sema6A^−/−^* brains were found in the dLGN, compared to 91.61 ± 3.17% of back-labeled thalamic cells in *Sema6A^+/−^* brains, *p* < 0.0001). Placements of DiA in parietal cortex in *Sema6A^−/−^* brains back-labeled VB cells, though this labeling was more medial within this nucleus than in *Sema6A^+/−^* animals (Figure 5C and 5D). Moreover, the areas back-labeled within VB by placements into occipital and parietal cortex were contiguous but showed minimal overlap. We did not observe any double-labeled cells.

Taken together, these data show that in *Sema6A^−/−^* embryos and neonates, axons of the dLGN specifically are misrouted to the surface of the ventral telencephalon, and the topography of thalamocortical projections from VB is expanded caudally into the unoccupied occipital cortex.

### Retinal Projections and Cortical Patterning Are Unaffected in *Sema6A* Mutants

Because *Sema6A* is also expressed in retinal ganglion cells, we wanted to test whether abnormal development of retinal projections in *Sema6A^−/−^* embryos might secondarily cause the defects observed in the projection of visual TCAs. We injected DiI and DiA crystals in the eye and V1, respectively, in wild-type and *Sema6A* mutant mice embryos at E16.5. In wild-type embryos (*n* = 3), retinogeniculate projections were observed in the ventral lateral geniculate nucleus (vLGN) and dLGN (Figure 5E and 5F). At the dLGN, this projection overlapped with back-labeled cells and cortical axons labeled from the visual cortical injections (Figure 5F). However, in *Sema6A^−/−^* embryos (*n* = 3/3), although the interconnectivity between visual cortex and thalamus was shifted medially, retinogeniculate projections still arrived to the vLGN and dLGN in a normal fashion (Figure 5G and 5H). Thus, the defects observed in the development of visual TCAs are not due to abnormal development of the projection from the retina or to abnormal differentiation of the LGN, which can still act as a specific target for retinal axons.

Similarly, to test whether changes in cortical patterning could account for the topographical defect observed in TCAs connectivity in *Sema6A^−/−^* mutants, we performed in situ hybridization of specific cortical area markers on both wild-type (*n* = 9) and *Sema6A^−/−^* (*n* = 8) brains at P0. In wild-type brains *EphA7* is expressed both rostrally and caudally in the cortex, but is largely absent from parietal cortex, whereas *EphrinA5* is expressed in a complementary pattern (Figure S3A and S3C). We did not find any changes in the expression of *EphA7* and *EphrinA5* in *Sema6A^−/−^* brains (Figure S3B and S3D). The expression of other cortical area markers (*Cad6*, *Cad8*, and *RZRβ*) was also unaltered in *Sema6A^−/−^* brains at P0 (unpublished data). At earlier (E14.5 and E16.5) and later (P7) developmental stages, there were also no differences observed in the expression of cortical area markers between wild-type and *Sema6A^−/−^* brains (unpublished data). These data indicate that intrinsic cortical patterning is unaltered in *Sema6A* mutants.

### Postnatal Recovery of Grossly Normal Thalamocortical Connectivity

**Figure 6:**
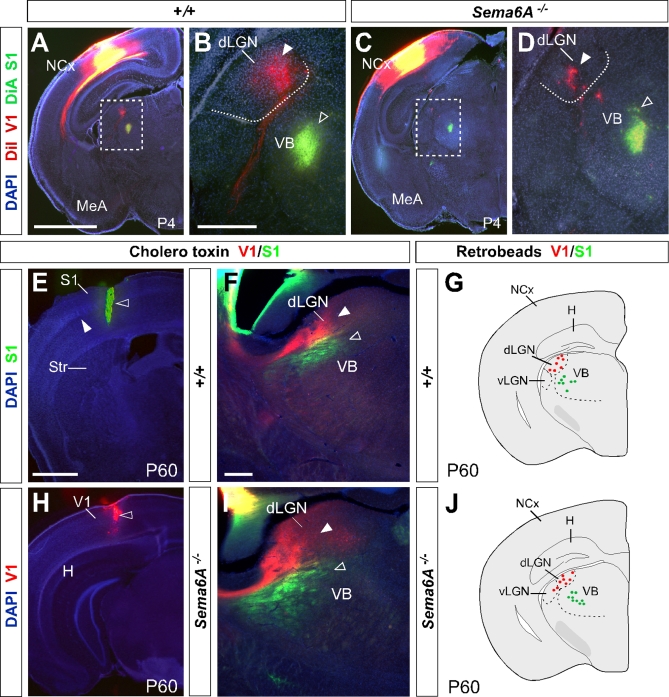
Postnatal Recovery of Visual Thalamocortical Connectivity in *Sema6A^−/−^* Mice All sections are coronal. (A and B) In wild-type brains, DiI and DiA crystal placement in occipital and parietal cortex, respectively, back-labeled cells in the dorsal lateral geniculate nucleus (dLGN; filled arrowhead) and the ventrobasal complex (VB) (open arrowhead) at P4. Dashed box indicates the area in (A) that is magnified in (B). (C and D) In contrast to what happened at earlier stages, in *Sema6A^−/−^* brains at P4, DiI crystal placement in the visual cortex back-labeled cells predominantly in the dLGN (filled arrowhead). DiA crystal placement in the somatosensory cortex back-labeled cells in the VB (open arrowhead). Dashed box indicates the area in (C) that is magnified in (D). (E, F, H, and I) Red and green cholera toxin (CT) was injected into the visual (V1, open arrowhead in [H]) and somatosensory (S1, open arrowhead in [E]) cortex, respectively, of wild-type and *Sema6A^−/−^* adult mice. In wild-type and *Sema6A^−/−^* mice, red CT back-labeled cells and axon terminals in the dLGN (filled arrowheads in [F and I]), and green CT back-labeled cells and axon terminal in the VB (open arrowheads in [F and I]). (G and J) Schematic diagrams showing the location of cell somata containing either red or green retrobeads, from injections in the visual (red) and somatosensory (green) cortices of adult wild-type and *Sema6A^−/−^* brains. In wild-type and *Sema6A^−/−^* brains, red retrobeads were found in the dLGN and never the VB in the dorsal thalamus. Green retrobeads were only ever observed in the VB. Scale bars in (A and C) indicate 1 mm; in (B and D) 500 μm; in (E and H) 1 mm; and in (F and I) 300 μm. MeA, medial amygdala; NCx, neocortex.

We investigated thalamocortical connectivity at P4 using multiple cortical dye placements. Small crystals of DiI and DiA were placed in the primary visual (V1) and primary somatosensory (S1) cortices, respectively, in wild-type and *Sema6A^−/−^* brains (Figure 6). In wild-type brains (*n* = 2/2), a DiI placement in V1 labeled thalamic cell bodies and cortical axons in the dLGN, whereas a DiA placement in S1 labeled cell bodies in the VB (Figure 6A and 6B). Interestingly, in *Sema6A^−/−^* brains (*n* = 2/2), a DiI placement in V1 mainly labeled thalamic cell bodies in the dLGN (Figures 6C, 6D, and 5J; 79.49 ± 25.3% of thalamic cells back-labeled from occipital cortex in P4 *Sema6A^−/−^* brains were found in the dLGN, compared to 99.49 ± 1.51% of back-labeled thalamic cells in wild-type brains, *p* = 0.0428), whereas far fewer cells were labeled in VB, in sharp contrast to results from similar tracing experiments at E16.5 and P0 (Figure 5K. In *Sema6A^−/−^* brains at P4, just 20.5 ± 25.3% of cells back-labeled from the occipital cortex were found in the VB, compared to 96.56 ± 4.68% and 83.65 ± 6.9% in *Sema6A^−/−^* brains at E16.5 and P0, respectively, *p* < 0.0001). A DiA placement in S1 labeled cell bodies in the VB as expected (Figure 6C and 6D). This apparent rapid recovery of the normal thalamocortical connectivity was observed despite a persistent misprojection of TCAs from the dLGN to the ventral telencephalon in *Sema6A^−/−^* brains at P4 (unpublished data). Although the topographical sorting of TCAs was apparently restored at this stage, fewer back-labeled cells were detected in the dLGN after visual cortical dye placements in *Sema6A^−/−^* compared to wild-type brains (Figure 6B and 6D).

To characterize the extent to which any shift in thalamocortical connectivity persists in the adult *Sema6A^−/−^* mouse, we performed in vivo stereotaxic tracing studies. We injected red and green cholera toxin (CT) dyes in V1 and S1, respectively, in wild-type (*n* = 6) and *Sema6A^−/−^* (*n* = 8) mice at P60 (Figure 6E, 6F, 6H, and 6I). At this adult stage, injections of red and green CT showed a normal topographical arrangement of the thalamocortical connectivity in *Sema6A^−/−^* brains (Figure 6I) when compared with wild-type brains (Figure 6F).

We also used two different colors of retrograde tracing microspheres, injected in V1 (red) and S1 (green) in wild-type or heterozygous (*n* = 5) and *Sema6A^−/−^* (*n* = 6) mice at P60. The back-labeled thalamic cells of each color were plotted in the corresponding thalamic nuclei, dLGN or VB, in wild-type (Figure 6G) and *Sema6A^−/−^* brains (Figure 6J). These data demonstrate that the early embryonic shift in TCAs connectivity, observed at E16.5 and P0, is partially recovered at P4 and totally compensated in the adult *Sema6A^−/−^* mouse.

### Recovery Pathways for Misrouted TCAs

**Figure 7:**
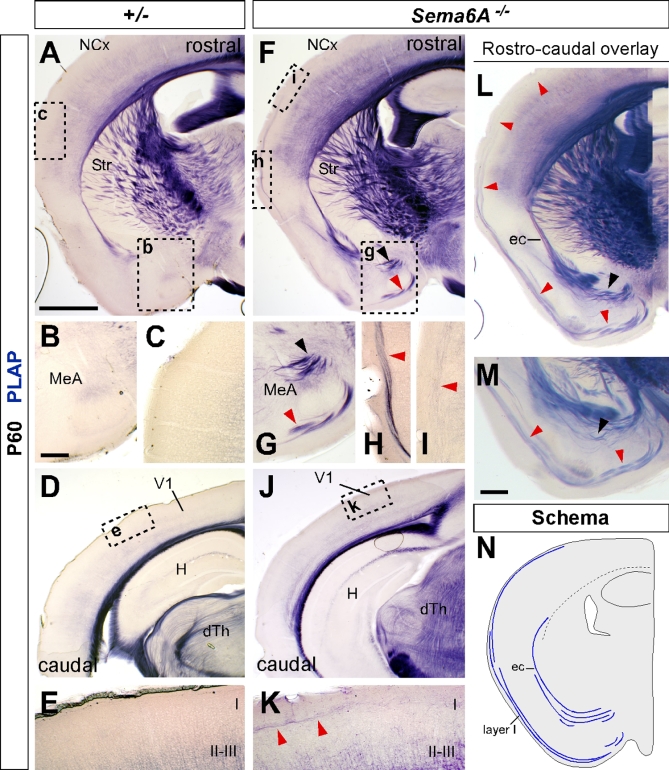
PLAP Staining Reveals Potential Recovery Pathways of Visual TCAs in *Sema6A^−/−^* Adult Brains All sections are coronal. (A and F) Low-power images of coronal sections of adult *Sema6A^+/−^* (A) and *Sema6A^−/−^* (F) brains, stained for PLAP. Misrouted TCAs are clearly visible in the ventral telencephalon of *Sema6A^−/−^* brains (arrowheads in [F]). (B, C, G–I) High-power view of these aberrant axon tracts (G), some of which project towards and into the external capsule, whereas others take a more ventral position and can be seen extending dorsally, close to the pial surface, up to the neocortex ([F, H, and I] red arrowheads). These axon tracts are never seen in wild-type or *Sema6A^+/−^* brains (B and C). (D, E, J, and K) Caudal coronal sections, showing the primary visual cortex (V1) of adult *Sema6A^+/−^* (D) and *Sema6A^−/−^* (J) brains. Note the presence of misrouted axons close to the pial surface of V1 in *Sema6A^−/−^* brains (red arrowheads in [K]). These axon tracts are never seen in wild-type or *Sema6A^+/−^* brains (E). (L and M). Overlaid serial sections through the same *Sema6A^−/−^* brain shown in (F) illustrates more clearly the persistent misrouted axons entering the external capsule (black arrowhead in [L and M]) or projecting more ventrally and close to the pial surface (red arrowheads in [L and M]) and up to the neocortex. (N) Schematic representation of the aberrant routes taken by misrouted TCAs to the neocortex in *Sema6A^−/−^* brains. Scale bars in (A, F, D, J, and L) indicate 1 mm; in (B, C, E, G, H, I and K) 200 μm; in (M) 500μm. I, layer I; II–III, layers II and III; dTh, dorsal thalamus; H, hippocampus; MeA, medial amygdala; NCx, neocortex; Str, striatum.

To investigate whether the misrouted TCAs from the dLGN persist in the adult mouse, we performed PLAP staining studies to reveal the pathway of Sema6A-positive axons in both *Sema6A* ^+/−^ (*n* = 4) and *Sema6A^−/−^* (*n* = 4) brains. Sema6A is expressed in oligodendrocytes in adults, and this staining thus labels all myelinated fibers. Surprisingly, in *Sema6A^−/−^* brains at P60, a misrouted bundle of axons was still observed at the ventral-most region of the telencephalon (Figure 7F, 7L, and 7M) in a similar location to the misrouted TCAs shown at earlier developmental stages. Similar ectopic bundles were never observed in any heterozygous adult brains (Figure 7A).

Interestingly, we could follow some PLAP-labeled axons up to the level of the visual cortex in *Sema6A^−/−^* brains (Figure 7H–7M). Misrouted labeled axons appear to follow one of two alternate routes: (1) they either turn laterally through the amygdala and join the external capsule, or (2) they continue to project ventrally and extend along the superficial margin of the telencephalon (Figure 7L–7N). In some cases, abnormal bundles could be followed either through the marginal zone or the external capsule up to the cortex (Figure 7L). At more caudal regions, misrouted axons can be seen close to the pial surface of the cortex, including the visual cortex, of *Sema6A^−/−^* brains (Figure 7K).

**Figure 8:**
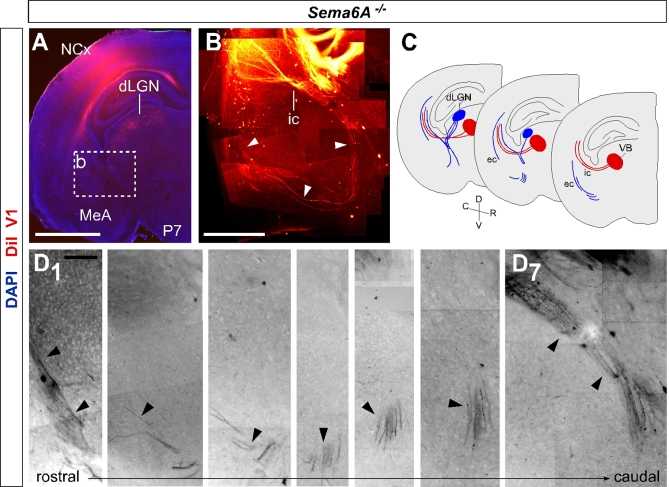
Recovery Pathways in *Sema6A^−/−^* Mouse Brains Revealed by Retrograde Labeling with DiI from the Primary Visual Cortex All sections are coronal. An injection of dissolved DiI in the V1 of *Sema6A^−/−^* mouse brains at P7 retrogradely labels cells bodies in the dLGN (A). The axons of these cells could be followed through consecutive sections extending ventrally into the ventral telencephalon and turning laterally and rostrally towards the external capsule (white arrowheads in [B]; black arrowheads in [D_1_–D_6_]). Many axons were also observed to initially project ventrally before turning to loop back to enter the internal capsule (black arrowheads in [D_7_]; schematic [C]). The entire pathway could be observed by overlaying consecutive coronal sections (B) as illustrated in the schematic diagram (C). Fibers back-labeled from V1 are labeled in blue. VB axons are not back-labeled from V1 at this stage, but their trajectories are shown for comparison in red. Scale bars in (A) indicate 2 mm, in (B) 500 μm. dLGN, dorsal lateral geniculate nucleus; ic, internal capsule; MeA, medial amygdala; NCx, neocortex; VB, ventrobasal complex.

To confirm the recovery pathways observed in PLAP-stained adult *Sema6A^−/−^* brains, a solution of DiI was injected into the primary visual cortex of wild-type or *Sema6A^−/−^* brains at P7. These injections back-labeled cell bodies in the dLGN specifically of both wild-type (*n* = 4; unpublished data) and *Sema6A^−/−^* brains (*n* = 3; Figure 8A). In wild-type animals, the axons of these back-labeled cells could be followed as they projected through the internal capsule to the cortex (unpublished data). In contrast, in *Sema6A^−/−^* brains, many labeled axons were observed projecting ventrally and rostrally in the ventral telencephalon, turning laterally to reach the external capsule at more rostral levels (Figure 8B–8D). Additionally, many axons were also seen to project in a more canonical fashion through the internal capsule, with some axons initially projecting ventrally before turning dorsally again to loop back and enter the internal capsule (Figure 8D_7_), suggesting that many initially misrouted dLGN axons may extend a branch at later stages and extend through the internal capsule to reach the cortex.

### Reduction in the Size of the dLGN and Visual Cortex in Adult *Sema6A^−/−^* Mice

**Figure 9:**
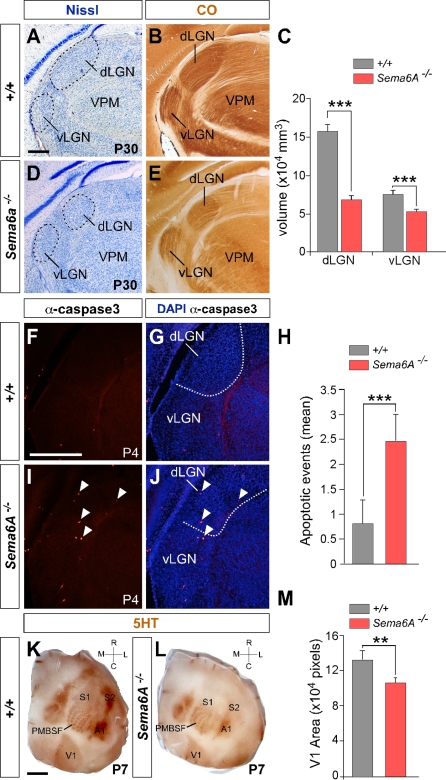
Effects of Loss of *Sema6A* on the Structure and Extension of Both dLGN and the Visual Cortex (A–E) Coronal sections of wild-type (A and B) and *Sema6A^−/−^* (D and E) adult mouse brains stained with Nissl (A and D) and cytochrome oxidase (B and E). Note the large reduction in size and volume of the dorsal lateral geniculate nucleus (dLGN) in *Sema6A^−/−^* brains (C). (F–J) Increased apoptosis in the dLGN in *Sema6A^−/−^* brains at P4 detected by caspase 3 antibody (filled arrowheads in [I and J]). (H) Graphical representation of the increased levels of apoptosis in the dLGN of *Sema6A^−/−^* compared to wild-type mice. (K and L) Tangential sections of wild-type (K) and *Sema6A^−/−^* (L) mouse brains stained for serotonin immunohistochemistry to reveal the cortical sensory domains at P7. Rostrocaudal and mediolateral directions are indicated by the cross-bars. Note that the size of the primary visual cortex (V1) in *Sema6A^−/−^* brains is reduced compared to that of wild-type brains. (M) Graphical representation of the area of the V1 in wild-type and *Sema6A^−/−^* brains at P7. Quantifications shown in (C, H, and M) are average + s.e.m. Double asterisks (**) indicate *p* < 0.001; triple asterisks (***) indicate *p* < 0.0005, *t*-test. Scale bars in (A, B, D, and E) indicate 300 μm; in (F, G, I, and J) 500 μm; and in (K and L) 2.5 mm. A1, primary auditory cortex; PMBSF, posteromedial barrel subfield; S1, primary somatosensory cortex; S2, secondary somatosensory cortex; vLGN, ventral lateral geniculate nucleus; VPM, ventroposterior medial nucleus.

To investigate whether the massive misrouting of TCAs during early embryonic stages has an impact on the development of the dLGN nucleus and visual cortex, we performed a series of histochemical studies in adult *Sema6A^−/−^* mice. Using both Nissl and cytochrome oxidase staining, we observed a significant reduction in the volume of the dLGN in *Sema6A^−/−^* brains (*n* = 4) compared with wild-type littermates (*n* = 4) at P30 (Figure 9A–9E). These data suggest that dLGN neurons whose axons do not reach their target on time die during development. To further test this possibility, we performed an immunostaining against a cleaved caspase-3 to detect apoptotic cells in the thalamus of wild-type and *Sema6A^−/−^* brains at P4. At this stage, we observed an almost 3-fold increase in the number of apoptotic cells in the dLGN of *Sema6A^−/−^* brains (*n* = 5) compared to controls (*n* = 6; Figure 9F–9J), suggesting that many of the dLGN neurons, whose axons were misrouted, do indeed die during development.

We next investigated whether the early lack of dLGN afferents to the visual cortex together with the transient invasion of somatosensory input to this cortical area might affect the final relative representation of cortical areas in *Sema6A^−/−^* adult mice. We examined the cortical area occupied by S1 and V1 in tangential sections stained for serotonin (5HT) immunoreactivity in wild-type (*n* = 5) and *Sema6A^−/−^* (*n* = 5) brains at P7. Although we observed no changes between wild-type and *Sema6A^−/−^* brains in the relative position of these cortical areas (Figure 9K and 9L), we observed a significant reduction in the size of V1 in *Sema6A^−/−^* mouse brains (Figure 9L and 9M). Moreover, we observed a consistent change in the shape of the V1 cortical domain in *Sema6A^−/−^* mouse brains compared to wild-type littermates (Figures 9K and 9L). No changes were observed in the position and dimensions of the barrel field in *Sema6A^−/−^* mice. Together, these results strongly suggest that the reduction in the size of dLGN in *Sema6A^−/−^* mice leads to a reduction in the size of V1.

## Discussion

Our study of the *Sema6A* mutants revealed an initial subcortical pathfinding defect of thalamic axons specifically from the dLGN. This results in expansion of somatosensory thalamic axons into presumptive visual cortex during embryonic stages. Due to the viability of these mutants, we were able to assess the secondary consequences of early misrouting of the visual axons on postnatal cortical specification and adult thalamocortical topography. Remarkably, many dLGN axons are able to find their way to visual cortex during early postnatal stages, following alternate routes, and can establish almost normal patterns of thalamocortical connectivity in the adult. The general implications of these findings for principles of thalamic axon guidance and cortical arealization are discussed below.

The failure of dLGN axons to arrive to the occipital cortex in *Sema6A^−/−^* brains at embryonic stages results in the dramatic expansion of the domain of VB axons into this region. Importantly, we observe no changes in cortical gene expression patterns at early stages, indicating that the removal of Sema6A from the cortex does not affect global cortical patterning. This early caudal shift of thalamocortical targeting has also been observed in the other mutants with misprojected dLGN axons (i.e., *Ebf1*, *Dlx1–2* double mutants [16]; reviewed in [2,18]). We observed back-labeling of VB from injections placed in occipital cortex, but did not observe a dramatic shift in connectivity from more rostral injection sites in parietal cortex (the normal position of S1), which still back-labeled VB (though more medially). This is more consistent with a caudal expansion of the innervation zone of VB axons in *Sema6A* mutants than with an overall shift of all thalamic connections.

A current model of thalamic axon pathfinding proposes an essential role for intermediate targets, in the ventral telencephalon (vTel), in guiding TCAs to specific cortical areas [2,17,18,20,29–31]. Mutations in a number of genes expressed predominantly in the vTel (*Ebf1*, *Dlx1/2*) affect thalamic projections subcortically in a manner that seems to be passively carried through in their projections to the cortex, at least at birth [16]. Analysis of the *Sema6A* mutants at postnatal stages reveals, however, that initially misrouted axons from the dLGN can eventually make appropriate connections to visual cortex. Remarkably, many of these projections seem to occur through alternate routes, either via the external capsule or a superficial route along the outside of the telencephalon. It is also possible that some projections are made via collaterals through the internal capsule that arise at later stages. These findings demonstrate that correct subcortical axonal sorting is not required for eventual projection to a specific cortical area and, further, that the normal temporal sequence of arrival of thalamic axons to the cortex is also not essential for correct targeting. In addition, they show that subcortical sorting is not sufficient to permanently determine connectivity as the initial shift in cortical targeting of VB axons that is apparent at embryonic stages can be corrected after birth. These conclusions are consistent with a growing body of research demonstrating the existence of cortical guidance cues for thalamic axon rearrangements [11,13,26,32] and suggest that the actions of these signals may be effective at a distance [22] to selectively attract misrouted dLGN axons to the appropriate cortical area. They also suggest that guidance cues within the neocortex exist not just in the subplate, but also across the developing cortical layers [26], allowing navigation even in the marginal zone, as demonstrated by the pathway follow by some of the misrouted LGN axons in the *Sema6a^−/−^* mutants.

The interpretation that subcortical sorting does not determine final cortical targeting would seem to be challenged by a number of other mutants that show early, global defects in subcortical thalamic projections, accompanied by later, highly specific defects in thalamocortical connectivity. For example, in double *Ephrin-A5*;*EphA4* mutants [30] and in mutants in either *CHL1* or *Npn1* [20], rostral thalamic axons project more caudally than normally both subcortically and up to the cortex itself at embryonic stages. In both cases, a defect in thalamocortical connectivity is also apparent at postnatal stages, involving excess connectivity of one thalamic nucleus with a particular cortical area, although it is much more selective, and differs between *Ephrin-A5*;*EphA4* and *CHL1* or *Npn1* mutants. In both cases, the early defect was interpreted as the cause of the later defect, but this has not been shown directly and the selective (and different) nature of the defects at later stages suggests that most of the early misrouting has in fact been corrected and that the postnatal connectivity defects are more likely to reflect later functions of these genes in the cortex itself.

Overall, these studies and our data are thus consistent with a model in which subcortical sorting of thalamic axons is coordinated with eventual cortical targeting, possibly using the same cues at both levels. However, subcortical targeting does not appear to be either strictly necessary or sufficient to determine final connectivity patterns as additional mechanisms exist to restore thalamocortical connectivity to a specific cortical area when alterations during embryonic development occur.

The recovery of the dLGN projection to visual cortex, in spite of previous occupation of this territory by VB axons suggests that dLGN axons have an advantage in the innervation of that particular cortical area. This must be in addition to selective axon guidance to this region as arrival of VB axons to this area is clearly not sufficient to enable them to make permanent connections, at least when faced with competition from later-arriving dLGN axons. A model to explain this would be that dLGN axons and presumptive visual cortex express some matching label(s) that confer this advantage. One candidate for such a cue is the neurotrophin NT-3, which is specifically required for dLGN axons to invade the cortical plate in V1 [33]. NT-3 has been shown to be most strongly expressed in presumptive visual cortex (V1) from around P0 [34], while its receptor TrkC, is selectively expressed by neurons in the dLGN. If such a matched cue is essential then VB axons that at early stages project into the subplate of occipital cortex may not be able to invade the cortical plate, allowing later-arriving dLGN axons to do so. Indeed, if the function of NT-3 in this context shares similarities with trophic signaling [35] then dLGN axons might actively secrete factors that promote withdrawal of VB axons. Axon–axon interactions mediated by surface receptors and cell adhesion molecules [36] might also actively mediate segregation of visual and somatosensory axons [37].

Activity-dependent mechanisms mediating the competitive advantage of dLGN axons for presumptive visual cortex must also be considered, especially as the process takes place during the first few postnatal days, by which time thalamic axons have normally entered into the cortex and formed fully functional synapses [32,38,39]. A number of studies have examined the potential role of electrical activity in areal targeting of thalamic axons. Intracranial infusion of the sodium channel blocker tetrodotoxin (TTX) caused dLGN axons to inappropriately innervate the subplate of cortical areas that they would normally bypass [40]. This could be taken as an instructive role for patterned activity in establishing areal connectivity but could alternatively be explained by an earlier effect of TTX on biochemical signaling pathways downstream of guidance receptors [41], or by feedback onto the expression levels of guidance molecules [42]. This interpretation is more consistent with the known specificity of thalamic axon targeting from the earliest stages [10,11,43] and the lack of effects in areal targeting observed in embryonic SNAP-25 mutants [39,44].

Finally, although our study demonstrates spectacular plasticity of thalamocortical connectivity during early postnatal life, there are some changes in the cortical architecture that persist into adulthood. The reduction in size and change in shape of V1 in *Sema6A* mutants, which are far more subtle than those observed in enucleation experiments [1,5,45,46], suggest that they may be an interesting model to study some less well-characterized processes, including the separation of the termination zones of primary thalamic axons into discrete areas, the innervation of intervening areas by axons from secondary nuclei, the formation of distinct borders and the hierarchical dependence of secondary and higher-order areas on correct specification of primary areas (reviewed in: [47,48]).

## Materials and Methods

### Animals.

All animal procedures were performed to relevant national and international licensing agreements and in accordance with institutional guidelines. *Sema6A* mutants were identified in a gene trap screen, as described previously [49]. Insertion of the gene trap vector pGT1PFS into intron 17 results in a fusion of upstream exons of Sema6A with TM-β-galactosidase-neomycin phosphotransferase. This fusion protein is sequestered intracellularly [28]. PLAP is cotranscribed but translated independently from an internal ribosome entry site. No wild-type transcripts are produced from this allele [28].

### Dye tracing studies.

Brains from E16.5 (*n* = 18), P0 (*n* = 45), P4 (*n* = 8), P7 (*n* = 6), and P30 (*n* = 26) were used in the study. To label thalamic and corticofugal fibers, single crystals of 1,1′-dioctadecyl-3,3,3′,3′-tetramethylindocarbocyanine perchlorate (DiI) and 4-(4-(dihexadecylamino) styryl)-N-methylpyridinium iodide (DiA) (Molecular Probes) were placed with a stainless steel electrode into the visual and somatosensory dorsal thalamic nuclei or the visual and somatosensory cerebral cortex of both hemispheres of each brain. After injections, brains were kept in 2% paraformaldehyde for between 3 wk and 2 mo at room temperature in the dark. Back-labeling of dLGN neurons and their axons in P7 animals was performed under hypothermia-induced anesthesia. A small incision was made in the scalp to reveal the skull, and a fine needle was used to pierce the skull above the primary visual cortex. A Hamilton syringe was used to inject 0.5 μl of a 10% solution of DiI in absolute ethanol, into the primary visual cortex. The scalp was bonded with tissue adhesive (Dermabond) and animals were allowed to survive for 24–48 h to allow for adequate retrograde labeling before being sacrificed. Dissected brains were postfixed for 24 h in 4% PFA at 4 °C. Brains were washed in PBS (0.1 M, pH 7.4), embedded into 4% agarose (Sigma), and cut at 100 μm with a Vibroslicer (Leica, VT1000S). Sections were counterstained with 2.5 μg/ml of bis-benzimide (Sigma) or with 0.5 μg/ml DAPI (4′-6-diamidino-2-phenylindole), mounted in PBS/glycerol or AquaPolymount (Polysciences) onto slides, and analyzed using an epifluorescence microscope (Leica, DMR, or Zeiss) and a laser scanning confocal microscope (Leica, DMRE).

### Histochemistry and immunohistochemistry.

Mice were perfused with 4% paraformaldehyde or a mixture of 1% paraformaldehyde/1.5% glutaraldehyde (for the cytochrome oxidase staining) in PBS. Brains were removed, postfixed in the same fixative overnight at 4 °C and embedded in 4% agarose. Serial 50- or 100-μm sections were cut on a vibratome (Leica; VT1000S) and processed for PLAP staining as previously described [28]. Alternatively, following perfusion, brains were postfixed in the same fixative for 3 h and cryoprotected with 30% sucrose in PBS. Serial 40-μm sections were cut on a freezing microtome and processed for Nissl staining (0.5% cresyl violet solution; *Sema6A^+/−^*: P0, *n* = 2; P30, *n* = 6, and *Sema6A^−/−^*: P0, *n* = 2; P30, *n* = 6). For cytochrome oxidase staining, cortical hemispheres were dissected from adult mice (*Sema6A^+/−^*, *n* = 9; *Sema6A^−/−^*, *n* = 9), postfixed between glass slides and cryoprotected before sectioning and processing. For immunohistochemistry, dissected brains were postfixed in 4% paraformaldehyde for 24 h, washed in PBS, embedded in 4% agarose, and sectioned (40–60 μm) on a vibratome. P 4 *Sema6A^+/+^* (*n* = 7) and *Sema6A^−/−^* (*n* = 5) mouse brains were treated for immunofluorescence with rabbit antibody to cleaved caspase-3 (1:200; Cell Signaling Technologies). Similarly, immunofluorescence with mouse antibody to neurofilament (1:100; DHSB) was detected on sections from E16.5 *Sema6A* ^+/+^ (*n* = 2) and *Sema6A^−/−^* (*n* = 2) mouse brains. Tangential sections were cut from flattened cortical hemispheres of P7 *Sema6A* ^+/+^ (*n* = 5) and *Sema6A* knockout (KO; *n* = 10) mouse brains and incubated with antibody to serotonin (1:50,000; Immunostar), which was then detected with biotinylated secondary antibodies using the Elite ABC kit (Vector). Results were documented using a digital camera (Leica DC500; Canon Powershot S40) or an epifluorescence microscope (Zeiss) and digital camera (Olympus), and the images compiled with Adobe Photoshop 8.0 or Adobe Photoshop CS software.

### Retrograde tracing with cholera toxin and fluorescent latex microspheres.

Green and red fluorescent latex microspheres (Lumaflor) were used to labeled axonal projection from somatosensory and visual cortical areas, respectively, to the corresponding thalamic nuclei in *Sema6A* ^+/+^ (*n* = 4) and *Sema6A^−/−^* (*n* = 4) mice. Animals were anesthetized with 2.7 mg/kg Hypnovel (Roche), Hypnorm (Janssen), and distilled H_2_O mixture (1:1:2 volume ratio), which was delivered intraperitoneally, and placed in a stereotaxis frame. After the skin was disinfected and incised, a microdrill was used to perform a craniotomy. Glass micropipettes (Clark Electromedical Instruments) and a binocular stereo-microscope (Zeiss) were used to inject a single injection of 0.3–1.0 μl of CT or microspheres into S1 or V1. Animals were allowed to survive for 24 to 48 h to permit adequate retrograde transport of the CT or microspheres to thalamic cell somata.

### In situ hybridization.

In situ hybridization was performed on 50-μm vibratome sections of E14.5 *Sema6A* (wild-type [WT]: *n* = 2, *Sema6A^+/−^* [HT]: *n* = 4 and KO: *n* = 4), P0 *Sema6A* (HT: *n* = 9, KO: *n* = 8), and P7 *Sema6A* (HT: *n* = 4, KO: *n* = 4) mouse brains, as previously described [50]. The following digoxigenin-labeled RNA probes were used: *Sema6a* (a gift from W. Snider); *EphA7*, *EphrinA5*, *Cadherin6*, and *RZRβ* (kindly provided by J. Rubenstein, with permission from the original researchers); *Cadherin8* (241–1,481 of mouse Cad8; GenBank accession number X95600; obtained by reverse transcription [RT]-PCR).

### Quantification.

The number of cells in the dLGN and the VB back-labeled from the occipital cortex were manually counted in consecutive 100-μm sections of E16.5 *Sema6A^+/−^* (*n* = 30 sections, 4 animals) and *Sema6A^−/−^* (*n* = 41 sections, 6 animals), P0 *Sema6A^+/−^* (*n* = 11 sections, 2 animals) and *Sema6A^−/−^* (*n* = 10 sections, 2 animals), and P4 wild-type (*n* = 9 sections, 2 animals) and *Sema6A^−/−^* (*n* = 12 sections, 2 animals) brains. The numbers of cells back-labeled to either the dLGN or VB for animals of a given age were compared using the Wilcoxon two-sample test and found to be significant at 99.9% confidence limits at E16.5 and P0. As the absolute number of cells back-labeled is dependent on the size of dye crystal used, we also analyzed the proportion of labeled cells in either the dLGN or VB of a given section. The proportional values were Arcsine transformed for statistical analysis by Wilcoxon two-sample tests. The area of the dLGN and vLGN thalamic nuclei was measured in 40-μm cytochrome oxidase serial sections from *Sema6A^+/−^* (*n* = 5) and *Sema6A^−/−^* (*n* = 4) brains using SigmaScan Pro software (SigmaScan). The volume of the dLGN and vLGN was calculated using the Cavalieri method. The relative area of V1 was measured in tangential sections of P7 of *Sema6A^+/−^* (*n* = 5) and *Sema6A^−/−^* (*n* = 10) brains, stained for serotonin immunohistochemistry, using Cell A software (Soft Image System).

## Supporting Information

Figure S1 PLAP Staining Reveals a Large Bundle of Misrouted TCAs at Caudal Levels in *Sema6A^−/−^* Brains at E16.5 (A–D) Rostral–caudal consecutive sections of PLAP-stained *Sema6A^−/−^* mouse brain at E16.5 reveals a large bundle of misrouted thalamocortical axons at more caudal levels. Whereas many labeled TCAs project normally through the internal capsule towards the cortex (black arrowheads in [A and B]), misrouted axons can be seen to project deep into the ventral telencephalon and project along its most superficial aspect (red arrowheads). Scale bars indicate 500 μm.
(1.44 MB TIF)

Figure S2 DiI and DiA Crystal Placements in the Ventral Telencephalon and Internal Capsule Confirm That dLGN Axons Do Not Enter the Internal Capsule in *Sema6A^−/−^* Brains at E17.5 Sections are at 45° to the coronal plane to encompass the internal capsule and dorsal thalamus.
(A and B) A DiA crystal placed in the internal capsule of wild-type brains at E17.5 broadly labels cells throughout the dorsal thalamus.
(C) In these same animals, a DiI crystal in the ventral telencephalon fails to label any cells in the dorsal thalamus, indicating that all TCAs project through the internal capsule in these animals.
(D–F) In *Sema6A^−/−^* brains at the same age (D and E), a DiA crystal in the internal capsule labels cell bodies in the VB but specifically not in the dLGN, whereas a DiI crystal in the ventral telencephalon does label dLGN neurons specifically, indicating that dLGN axons in these animals do not project at all to the internal capsule at this age (F).
Scale bars in (A–D) indicate 500 μm.
(1.90 MB TIF)

Figure S3 Cortical Area Markers Show No Defect in the Relative Position of Principal Cortical Areas in *Sema6A^−/−^* Brains at P0 (A and D) In situ hybridization with DIG-labeled probes for *EphA7* (A and C) and *EphrinA5* (B and D) on sagittal sections of wild-type (A and B) and *Sema6A^−/−^* (C and D) brains. *EphA7* is expressed in the rostral and occipital neocortex (NCx), but is absent from the putative somatosensory cortex. In contrast, *EphrinA5* is expressed is highly expressed in the somatosensory cortex. dTh, dorsal thalamus. Scale bars in (A–D) indicate 500 μm.
(1.17 MB TIF)

## Abbreviations

CT: cholera toxin
dLGN: dorsal lateral geniculate nucleus
E: embryonic day
P: postnatal day
PLAP: placental alkaline phosphatase
S1: primary somatosensory cortex
TCA: thalamocortical axon
V1: primary visual cortex
VB: ventrobasal
vLGN: ventral lateral geniculate nucleus
