# Supplementary figures and images for: Specificity and Plasticity of Thalamocortical Connections in Sema6A Mutant Mice
Source: PLoS Biol. 2009 Apr 28;7(4):e1000098. doi: 10.1371/journal.pbio.1000098 (PMC2672616; doi:10.1371/journal.pbio.1000098)

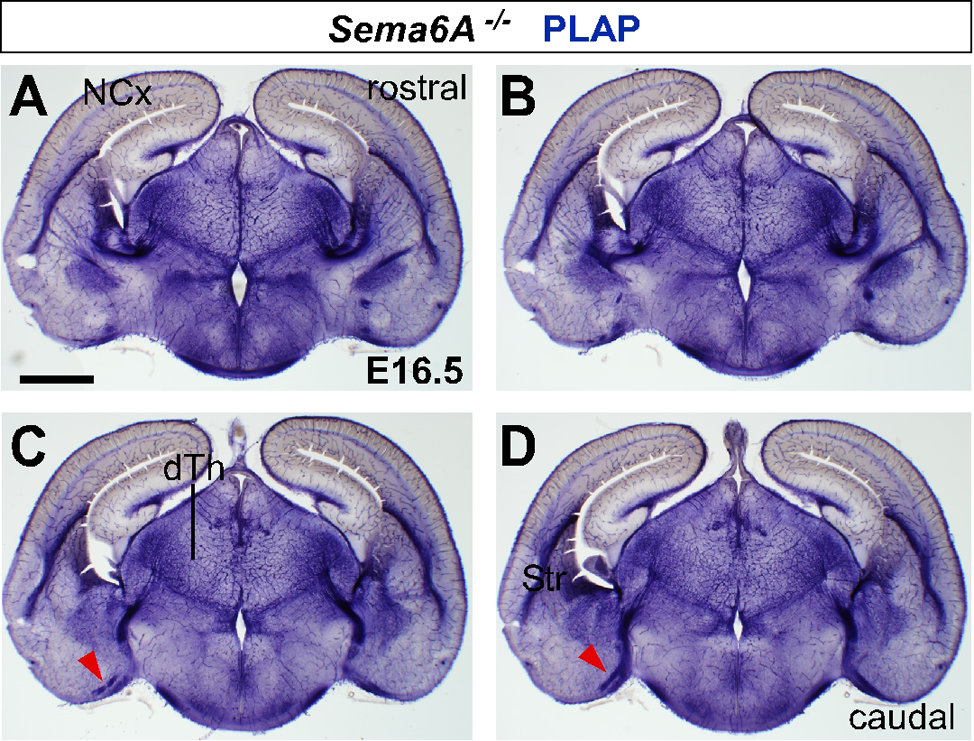

Supplement: Figure S1 — (A–D) Rostral–caudal consecutive sections of PLAP-stained Sema6A−/− mouse brain at E16.5 reveals a large bundle of misrouted thalamocortical axons at more caudal levels. Whereas many labeled TCAs project normally through the internal capsule towards the cortex (black arrowheads in [A and B]), misrouted axons can be seen to project deep into the ventral telencephalon and project along its most superficial aspect (red arrowheads). Scale bars indicate 500 μm. (1.44 MB TIF) [file pbio.1000098.sg001.tif]

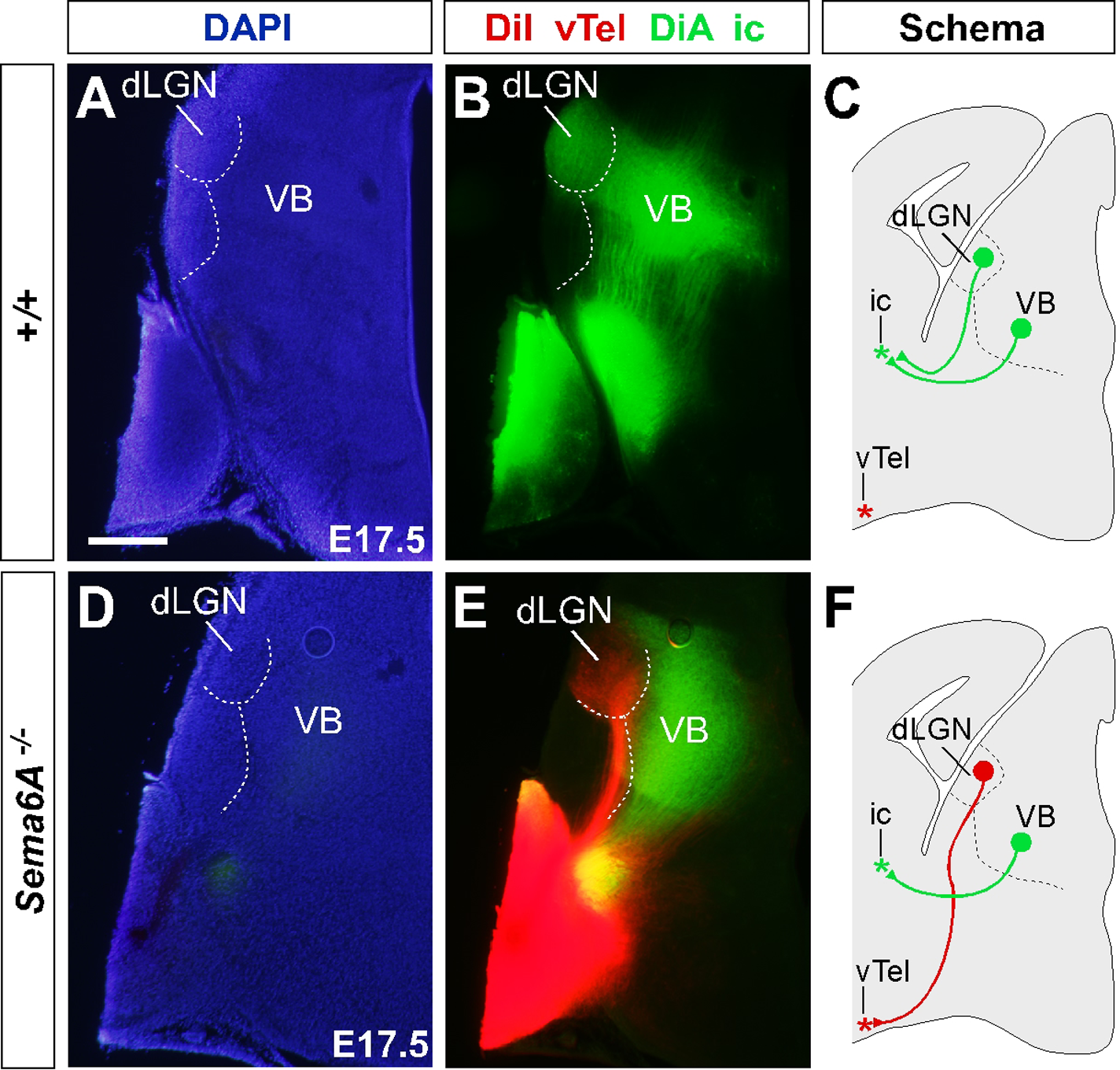

Supplement: Figure S2 — Sections are at 45° to the coronal plane to encompass the internal capsule and dorsal thalamus. (A and B) A DiA crystal placed in the internal capsule of wild-type brains at E17.5 broadly labels cells throughout the dorsal thalamus. (C) In these same animals, a DiI crystal in the ventral telencephalon fails to label any cells in the dorsal thalamus, indicating that all TCAs project through the internal capsule in these animals. (D–F) In Sema6A−/− brains at the same age (D and E), a DiA crystal in the internal capsule labels cell bodies in the VB but specifically not in the dLGN, whereas a DiI crystal in the ventral telencephalon does label dLGN neurons specifically, indicating that dLGN axons in these animals do not project at all to the internal capsule at this age (F). Scale bars in (A–D) indicate 500 μm. (1.90 MB TIF) [file pbio.1000098.sg002.tif]

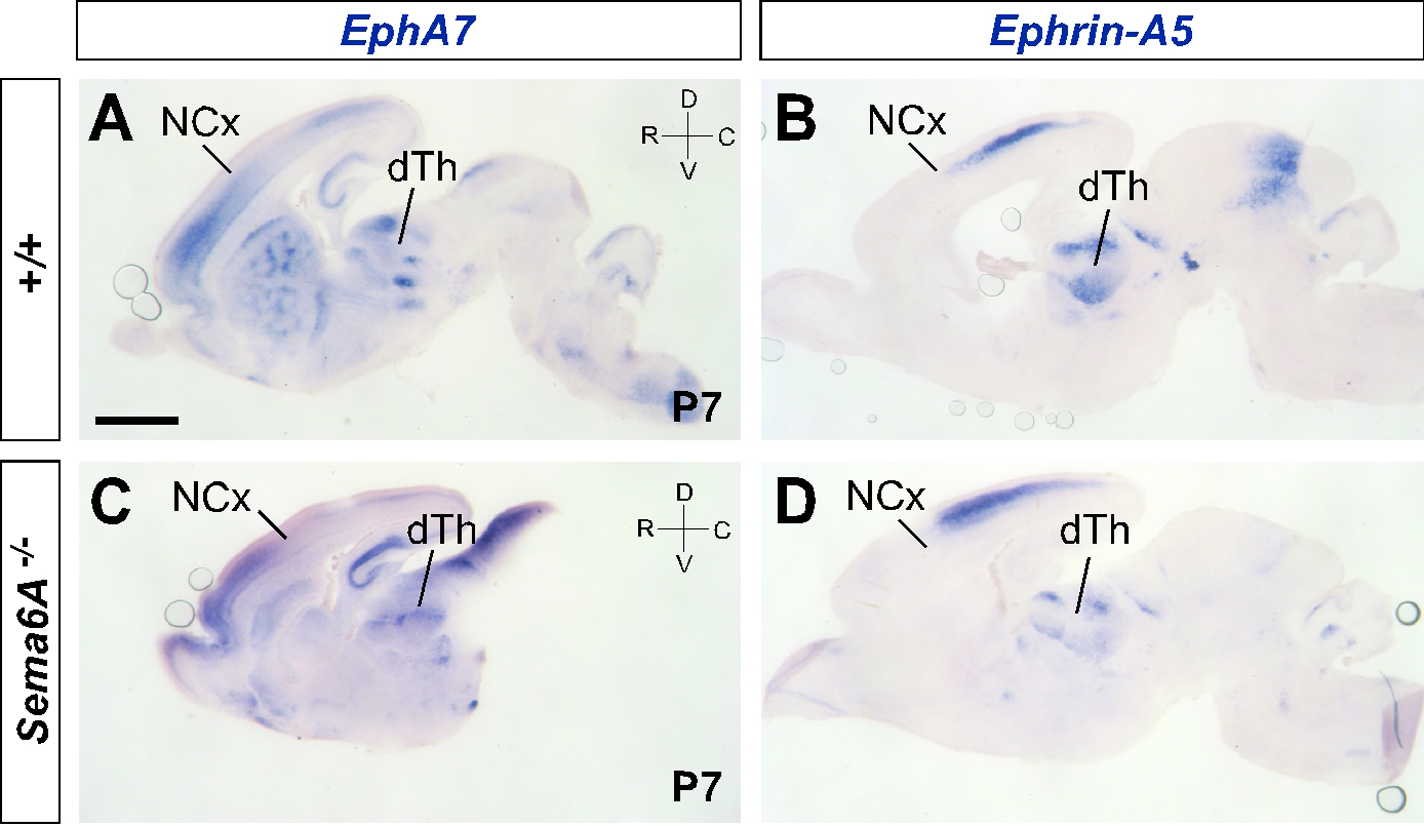

Supplement: Figure S3 — (A and D) In situ hybridization with DIG-labeled probes for EphA7 (A and C) and EphrinA5 (B and D) on sagittal sections of wild-type (A and B) and Sema6A−/− (C and D) brains. EphA7 is expressed in the rostral and occipital neocortex (NCx), but is absent from the putative somatosensory cortex. In contrast, EphrinA5 is expressed is highly expressed in the somatosensory cortex. dTh, dorsal thalamus. Scale bars in (A–D) indicate 500 μm. (1.17 MB TIF) [file pbio.1000098.sg003.tif]
